# Blood loss predictive factors and transfusion practice during percutaneous nephrolithotomy of kidney stones: a prospective study

**DOI:** 10.12688/f1000research.8993.1

**Published:** 2016-06-30

**Authors:** Firtantyo Adi Syahputra, Ponco Birowo, Nur Rasyid, Faisal Abdi Matondang, Endrika Noviandrini, Maruto Harjanggi Huseini

**Affiliations:** 1 Department of Urology, Cipto Mangunkusumo Hospital / Faculty of Medicine, Universitas Indonesia, Jakarta, 10430, Indonesia

**Keywords:** Bleeding, nephrolithiasis, PCNL, transfusion

## Abstract

**Objectives**

Bleeding is the most common complication of percutaneous nephrolithotomy (PCNL). Injudicious transfusion is frequently performed in current practice, even though it is not always needed. This study aimed to identify the predictive factors of blood loss in the PCNL procedure and evaluate the perioperative transfusion practice.

**Methods**

A prospective study of PCNL was randomly performed by two consultants of endo-urology at our institution. The inclusion criteria were adults with kidney pelvic stones >20 mm or stone in inferior calyx >10 mm or staghorn stone. Those with coagulopathy, under anti-coagulant treatment or open conversion were excluded. A full blood count was taken at baseline and during 12, 24, 36, 72-hours post-operatively. Factors such as stone burden, sex, body surface area, shifting of hematocrit level and amount of blood transfused were analyzed statistically using line regression to identify the predictive factors of total blood loss (TBL).

** **

** Results**

Eighty-five patients were enrolled in this study. Mean TBL was 560.92 ± 428.43 mL for both endo-urology surgeons. Stone burden was the most influential factor for TBL (p=0.037). Our results revealed that TBL (mL) = -153.379 + 0.229 × stone burden (mm2) + 0.203 x baseline serum hematocrit (%); thus considerably predicted the need for blood transfusion. A total of 87.1% patients did not receive perioperative transfusion, 3.5% received intra-operative transfusion, 7.1% received post-operative transfusion, 23% had both intra and post-operative transfusion, resulting in a cross-matched transfusion ratio of 7.72. Mean perioperative blood transfused was 356.00 ± 145.88 mL.

## Introduction

Kidney stones prove to be a common affliction in many countries worldwide because of high incidence and prevalence. In America, kidney stone incidence was found in 116 out of 100,000 individuals ^1^. A higher incidence was discovered in the German population aged 14 years and older, amounting to 720 out of 100,000 individuals ^2^. An excessively high number of cases was found in Asian countries, namely, 114.3 per 100,000 in Japan, while Iranian urolithiasis incidence was assessed at 145.1 in 2005 ^3^. A global increase in kidney stone cases was determined in individuals of all ages, sex, and races ^4^. In our institution we have, to date, treated an increasing number of kidney stone patients, from 182 in 1997 to 847 in 2002 ^5^.

Percutaneous nephrolithotomy (PCNL) is a urological minimally-invasive procedure to extract kidney stones by means of percutaneous access ^6^. Nowadays, PCNL is widely accepted to treat those complex kidney stone cases that are hard in consistency, of a large size, infected, obstructed with anatomical abnormalities, generally cases that could not be treated by other modalities ^7^. In European guidelines, PCNL is favoured as the treatment of choice for calyx and pelvic stones >20 mm ^2^ ^8^. For stones >20 mm, PCNL demonstrated a stone-free rate up to 78–95% ^9^. There has been a decrease in open surgery number in our institution during 2001–2009, whereby the PCNL procedure has become more frequent ^10^.

One of the most bothersome complications of PCNL is hemorrhage. Direct access to the pelvicalyceal system and intrarenal manipulation during PCNL procedures cause injury to the renal vasculature, particularly to the segmental and interlobar arteries. The renal-collecting system is rich in vascularization, covering 20% of the total cardiac output, and often results in hemorrhage during PCNL ^11^. The high percentage of blood loss and the necessity of transfusion often results in the erroneous management of hemorrhage during the PCNL procedure.

It has been reported that 1–11% of patients who underwent PCNL required blood transfusion; a higher transfusion rate, 2–53% was determined in the staghorn cases ^11^. Previous studies describe many hemorrhage risk factors during PCNL, such as, age ^12^, pre-operative urinary tract infections ^12^, large stones (exceeding 1250 mm ^2^) ^13^, staghorn calculi ^13, 14^, multiple access ^11, 13, 14^, diabetes mellitus ^11, 13, 15^, prolonged surgery time ^11– 13^ and stone composition ^13^. Other risk factors have been postulated: i.e. stone location, pre-operative hemoglobin, hydronephrosis grade, renal parenchymal thickness, however, to date, have not yet been proven. Many of the hemorrhage cases during PCNL could be managed conservatively, however, 0.8% patients required a more invasive procedure to deal with the bleeding ^14^.

To date there are no specific data available to determine the blood transfusion requirement during PCNL procedures. In our institution, blood units are requested pre-operatively according to clinical estimation. However, this does not always concede to the intra-operative blood loss. This study was aimed at predicting the amount of blood loss during PCNL by identifying the pre-operative factors that could possibly lead to a lower morbidity rate.

## Materials and methods

The present study includes patients who underwent percutaneous nephrolithotomy procedure in our hospital from October 2012 to October 2013. Adult patients (≥18 years old) with pelvic stones >20 mm, inferior calyx stones >10 mm or staghorn stones who agreed to enroll by written informed consent were included in this study. Those with coagulopathy, under anti-coagulant treatment or conversion to open procedure were excluded.

Patients were admitted the day prior to procedure. Stone burden was assessed pre-operatively by multiplying sum of length and width by means of imaging. The PCNL was randomly performed under spinal anesthesia by two endo-urology consultants. The patient was placed in prone position, access gained to pelvicalyceal system with fluoroscopic guidance, followed by dilatation using a metal and fascia dilator before application of sheath. The number and location of punctures were decided intra-operatively, based on pelvicalyceal system. We used pneumatic lithotripsy to break the stone which was subsequently extracted by forceps or grasper. The procedure was completed when the patient was stone-free and any arising complications alleviated.

Full blood counts were taken prior to procedure and thereafter at 12, 24, 36, 72-hours post-operatively. Total blood loss was calculated considering body surface area, sex-adjusted estimated blood volume, initial hematocrit level and 72-hour post-operative hematocrit level. Study protocol has been approved by the Ethical Committee, Faculty of Medicine, Universitas Indonesia (No.89/H2.F1/ETIK/2013).

We compiled a chart ^15, 16^ to assess total blood loss (TBL) to include sex, body surface area, shifting of hematocrit level and amount of blood transfused, as shown in Table 1.

**Table 1.** Operational definition.

Variable	Formula
Body Surface Area (m ^2^) ^15^	0.0235 × [Height (cm)] ^0.42246^ × [BW (kg)] ^0.51456^
Estimated Blood Volume (mL) ^15^	Female: [Body Surface Area (m ^2^)] × 2430
	Male: [Body Surface Area (m ^2^)] × 2530
Initial RBC (mL) ^15^	[Estimated Blood Volume (mL)] × [Initial Hematocrit level 24-hour pre-op (%)]
Final RBC (mL) ^15^	[Estimated Blood Volume (mL)] ×[Final Hematocrit level 72-hour post-op (%)]
Uncompensated RBC Loss (mL) ^15^	[Initial RBC (mL)] – [Final RBC (mL)]
Compensated RBC Loss (mL) ^15^	[Amount of RBC transfused from intra-op to 72-hour post-op]
Total RBC Loss (mL) ^15^	[Uncompensated RBC Loss (mL)] + [Compensated RBC Loss (mL)]
Total Blood Loss (mL) ^16^	$\frac{\left[\text{Total}\;\text{RBC}\;\text{Loss}\;(\text{mL})\right]}{\left[1/2\times (\text{Hematocrit}\;24-\text{hour}\;\text{pre-op}+\text{Hematocrit}\;72-\text{hour}\;\text{post-op})\right]}$
Cross match transfusion ratio/CT ratio ^17^	Amount of blood unit cross-matched : Amount of blood unit transfused
Under-transfusion ^18^	Defined as blood volume transfused deficit less than 15.0% of total blood loss (mL)
Over-transfusion ^18^	Defined as blood volume transfused excess greater than 15.0% of total blood loss (mL)

**Abbreviations:** RBC = red blood cell, BW = body weight

Bivariate analysis was done by correlating numerical variables with total blood loss, and associating categorical variables with perioperative blood transfusion amount. Those with significancy of <0.25 were further analyzed with multivariate analysis of linier and logistic regression.

## Results

A total of 85 PCNL procedures were performed on 85 patients (46 males, 39 females) who completed this study, thus gave statistical power of 0.8. The average age was 50.96 ± 11.87 years. Most of the patients complained of flank pain at initial presentation ( Table 2). Staghorn calculi were found in 50.6% patients.

**Table 2.** Patient Characteristics (n=85).

Variable		Number (Percentages)	
Sex	Male	46 (54.1%)
	Female	39 (45.9%)
Symptoms	Renal colic	17 (20.2%)
	Flank pain	76 (89.3%)
	Passing stone	29 (34.5%)
	Sandy urination	10 (11.9%)
	Haematuria	18 (21.4%)
	Dysuria	3 (3.6%)
	Fever	4 (4.8%)
	Urinary retention	2 (2.4%)
	Intermittency	2 (2.4%)
Stone Side	Right	50 (59.0%)
	Left	35 (41.0%)

The mean hematocrit drop was 5.20 ± 3.36%. Average total blood loss was 560.92 ± 428.43 mL with median 511.46 (95% CI: 0.00-1974.84) mL. There were two cases with pelvicalyceal laceration, one with massive hemorrhage (perioperative blood loss 1974.84 mL). There was no significant difference of total blood loss between the cases performed by the two PCNL surgeons (p>0.05).

Stepwise multivariate regression analysis which included variables with p-value < 0.25 ( Table 3) showed that stone burden was the most influential predictor of blood loss (p=0.037). Meanwhile operative time was found not associated (p-value >0.05) with blood loss. We assessed total blood loss (in mL) as -153.379 + 0.229 × stone burden (mm ^2^) + 0.203 × serum hematocrit baseline (%). This means it is predicted that a kidney stone patient with 1000 mm ^2^ stone burden and baseline hematocrit 40% will have 83.74 mL blood loss during PCNL procedure perioperative.

**Table 3.** Bivariate analysis between related factors and total blood loss.

Variable	p-Value	R
Stone burden**	0.067	0.200
Number of stones**	0.380	0.096
Serum creatinine baseline**	0.549	0.066
Red blood cell count**	0.095	0.182
Serum hematocrit baseline*	0.135	0.163
Serum hemoglobin baseline**	0.501	0.074
Body mass index**	0.298	0.114
Age*	0.670	0.047

*Pearson analysis **Spearman analysis

The average amount of blood units 435.29 ± 114.13 mL was cross-matched for each procedure pre-operatively ( Table 4). Nevertheless, 87.1% of patients did not receive blood transfusion perioperatively, thus yielding the blood transfusion rate as 12.9%. Blood transfusion was required by 3.5% patients intra-operatively, 7.1% post-operatively and 2.3% both intra and post-operatively. In total, the cross-matched transfusion ratio was 7.72. The average amount of blood transfused during PCNL procedure: 356.00 ± 145.88 mL.

**Table 4.** Blood transfusion pattern.

Blood Transfusion Proportion (%)		Average amount of blood unit cross- matched	Average amount of blood transfused (Intra-op – 72-hour post-op)	>Under- transfusion (%)	>Over- transfusion(%)
No transfusion	87.1%	435.29 ± 114.13 mL	356.00 ± 145.88 mL	0%	100%
Intra-operative transfusion	3.5%	***Cross Match Transfusion Ratio***			
Post-operative transfusion	7.1%	7.72			
Both intra & post-operative transfusion	2.3%				

Raw data for Table 3 of 'Blood loss predictive factors and transfusion practice during percutaneous nephrolithotomy of kidney stones' The raw data of the bivariate analysis between related factors and total blood loss are provided. Click here for additional data file. Copyright: © 2016 Syahputra FA et al. 2016 Data associated with the article are available under the terms of the Creative Commons Zero "No rights reserved" data waiver (CC0 1.0 Public domain dedication).

Raw data for Table 4 of 'Blood loss predictive factors and transfusion practice during percutaneous nephrolithotomy of kidney stones' The raw data for ‘Blood transfusion practice’ are provided. Click here for additional data file. Copyright: © 2016 Syahputra FA et al. 2016 Data associated with the article are available under the terms of the Creative Commons Zero "No rights reserved" data waiver (CC0 1.0 Public domain dedication).

## Discussion

In this study we did not use conventional, visual estimated blood loss to determine hemorrhage due to high bias factors, subjectivity, persistence of dilution effect and poor accuracy ^19– 23^. Laboratory parameters in our study were recorded until 72-hours post-operatively, in order to minimize intravenous hydration and retroperitoneal fluid absorption effects. We used the hematocrit level as the main parameter to determine blood loss rather than hemoglobin to avoid hemodilution effect ^15, 24^; Furthermore, the hematocrit level positively correlated with the total blood volume ^25^. It has been reported that also a center in Turkey applies a blood transfusion policy that depends on the hematocrit level (transfusion was indicated when hematocrit level was less than 30%) ^14^.

Stone burden was the most influential predictive factor for blood loss during the PCNL procedure in this study, similar to other studies performed. A multivariate analysis showed that complete and partial staghorn calculi were associated with a greater blood loss than with the calyx stones ^14^. Other studies concluded that larger stone burdens ^11^ or staghorn calculi ^26^ required a greater amount of unit blood transfused during the PCNL procedure compared to the smaller stones. Greater hematocrit level changes in staghorn calculi were found during PCNL, while further multivariate tests concluded that staghorn calculi were associated with a greater amount of blood loss (OR 1.92) and a greater decrease in the hemoglobin level compared to non-staghorn cases ^13^. Prolonged and excessive intra-renal maneuver performed for large stone burdens was assumed to increase incidence of injury to renal vasculature ^11^.

Our transfusion rate was similar to the one reported in a retrospective study from Pakistan showing an overall blood transfusion rate of 14.2% with one angioembolization performed to control hemorrhage ^26^. In our study, all cases presenting with massive hemorrhage could be managed conservatively. Lower blood transfusion rates were reported in two other studies from Pakistan, one study from United Kingdom and one study from the United States; these differences occurred due to the younger age group ^27^, supine position used ^28^ and balloon dilatation ^29, 30^. A higher transfusion rate (23.8%) compared to our result was shown in a retrospective analysis. An aggressive approach by torqueing the rigid nephroscope to maximize stone clearance at one stage was explained ^14^.

A precise estimation of surgical blood loss is essential in order to avoid excessive usage of blood units. Most of the previous studies emphasize the estimated blood loss rather than the objective prediction. Our calculation of total blood loss included perioperative factors: the patient’s blood volume (based on sex, body weight and height), the number of red cell units transfused, the hematocrit changes, and the amount of hemodilution. To our knowledge, this is the first study that has applied a mathematical approach to predict blood loss during PCNL procedures. We did not include hydronephrosis grading, parenchymal thickness and stone composition to analyze the predictive factors of blood loss, due to the possible limitations relating to our study.

## Conclusions

Stone burden was the most influential PCNL blood loss predictive factor in our institution. We estimated that the amount of blood requested and cross-matched for PCNL is much greater than the actual blood loss. Our principle was proposed as a guidance to reduce any unnecessary costs and excessive requirements of blood units.

## Consent

Written informed consent to participate in the study and publish clinical data was obtained by the patients.

## Data availability

The data referenced by this article are under copyright with the following copyright statement: Copyright: © 2016 Syahputra FA et al.

Data associated with the article are available under the terms of the Creative Commons Zero "No rights reserved" data waiver (CC0 1.0 Public domain dedication). 

F1000Research: Dataset 1. Raw data for Table 3 of 'Blood loss predictive factors and transfusion practice during percutaneous nephrolithotomy of kidney stones', 10.5256/f1000research.8993.d127629 ^31^

F1000Research: Dataset 2. Raw data for Table 4 of 'Blood loss predictive factors and transfusion practice during percutaneous nephrolithotomy of kidney stones', 10.5256/f1000research.8993.d127630 ^32^
