# Supplementary material for: Blood loss predictive factors and transfusion practice during percutaneous nephrolithotomy of kidney stones: a prospective study
Source: F1000Res. 2016 Jun 30;5:1550. [Version 1] doi: 10.12688/f1000research.8993.1 (PMC4943294; doi:10.12688/f1000research.8993.1)
Supplement: Peer review history

# Peer review: Referee response for version 1

**DOI:** 10.5256/f1000research.9674.r14725

**Published:** 2016-07-12

**Authors:** Doddy Musbadianto Soebadi

**Affiliations:** 1 Department of Urology, Airlangga University, Surabaya, Indonesia

This is a nice article concerning blood loss during percutaneous nephrolithotomy (PCNL) procedures. We need these data to confirm that this procedure is safe to be done in a proper way.

 The authors have meticulously analyzed and discussed the results.

I have read this submission. I believe that I have an appropriate level of expertise to confirm that it is of an acceptable scientific standard.

# Peer review: Referee response for version 1

**DOI:** 10.5256/f1000research.9674.r14722

**Published:** 2016-07-11

**Authors:** Manint Usawachintachit

**Affiliations:** 1 Department of Urology, University of California San Francisco, San Francisco, CA, USA; 2 Division of Urology, Faculty of Medicine, Chulalongkorn University and King Chulalongkorn Memorial Hospital, The Thai Red Cross Society, Bangkok, Thailand

The authors present a study looking for predictive factor of blood loss following PCNL from a single institution for 85 patients over a 1-year period. It’s interesting data in that while most relevant published studies were carried out retrospectively, this study is prospective. The primary endpoints were changes in hematocrit level and blood transfusion requirement over an early postoperative period.   

 Overall, the paper was written concisely with a good methodology to track blood loss level. I would recommend some revision enumerated below:  

**General comments**

- Besides grammatical errors, there are some misspelled words.

 

**Abstract**

- In the Result, a percentage of intra and postoperative transfusion should be 2.3%, not 23%.
- It may be helpful to add a conclusion to the abstract.  

 

**Introduction**

- No specific comments.

 

**Material and Methods**

- Were all patients included in this study consecutively?
- How was stone burden assessed by means of imaging? Was it measured from CT-scan, plain film, or IVP?
- How was a hematocrit level measured? Was it from a full blood draw or just fingertip needle sticks crude measurement?  
- What was an algorithm of decision for perioperative blood transfusion in this study? Did the authors make a judgement based on a “specific” cutoff point of the most recent blood count (at 12, 24, 36, and 72 hours after PCNL) or make a decision subjectively? There should be a systematic methodology in this issue because it significantly affected transfusion rate and volume.    

 

**Results**

- How many patients were excluded from this study because of a conversion to open procedure? Were they converted from profound intraoperative bleeding?
- What were a mean BMI of patients, and distribution of hydronephrosis in this series? The authors have mentioned in the Introduction that they were risk factors for blood transfusion flowing PCNL.
- What was a distribution of renal access (upper/middle/lower/multi-tract) in this study? Did number of renal access tract affect blood loss or transfusion rate?
- In a multivariate analysis of variables and blood loss, was stone burden (p = 0.037) the only one factor that remained statistically significant? This data was not shown in Table 3. What were other variables included in the multivariate analysis and why did the authors include operative time (p > 0.05) in this model?   

 

**Discussion**

- The authors mentioned a hematocrit cut-point of 30% from a Turkish study. Did the authors use the same cut-point for transfusion in this study?
- Transfusion rate in this series was relatively high (12.9%) and the authors have demonstrated a significant correlation between stone burden and blood loss. Could this high transfusion rate cause by a larger stone burden? This data (mean stone burden) was not shown in the manuscript.
- What is a clinical benefit of the mathematical equation to predict blood loss preoperatively? Could we use it to obviate blood request in some patients?

 

**Miscellaneous**

- In Table 2, it may be more understandable to order presenting symptoms by percentages.
- In Dataset 1, why did the authors perform PCNL in some stones of 5 mm ^2^ size. Is it more reasonable to treat them with ESWL or flexible ureteroscopy.  

I have read this submission. I believe that I have an appropriate level of expertise to confirm that it is of an acceptable scientific standard, however I have significant reservations, as outlined above.

# Peer review: Referee response for version 1

**DOI:** 10.5256/f1000research.9674.r14721

**Published:** 2016-06-30

**Authors:** M Hammad Ather

**Affiliations:** 1 Section of Urology, Aga khan University, Karachi, Pakistan

Authors have assessed the predictive value of various parameters for transfusion during / following PCNL. It is an important area as bleeding and need for transfusion is still a major concern during PCNL. I read with interest the fact that the authors have performed all procedures under spinal anesthesia. Although it is feasible and has been reported in literature it is both cumbersome for patients, anesthetist and surgeon to perform this procedure in an awake patient. In addition to maintain level of spinal anesthesia to provide pain free procedure and yet not compromising ventilation in a spontaneously breathing patient is a difficult task. It is not clear from the methods described if the procedure was performed in prone or supine position. In the methods section authors have noted that "the procedure was randomly performed.....", I am not sure as to what the authors intended to mean. In addition in the methods section authors have noted that the "The procedure was completed when the patient was stone-free and any arising complications alleviated. " the meaning of this sentence is also not clear. In the results section authors have noted that "statistical power of 0.8 ", kindly clarify the sentence.

 Authors noted stone burden as clinical (statistically significant) factor in predicting need for transfusion. This is conformed by many previous studies and authors conclusion does not surprise me.

 In recent years many scoring systems have been developed to predict complexity of the procedure and stone free rate like STONE nephrolithomeetry score, Guys score etc. Authors should comment why they decided to use stone burden rather than these scores in predicting complexity level of the procedure and predicting need for transfusion.

 A recent report by Un *et al.,* 2015 ^1^ noted transfusion requirement of ~10% and need for embolization in <1% in over 1400 PCNL over a 7 years period.

I have read this submission. I believe that I have an appropriate level of expertise to confirm that it is of an acceptable scientific standard, however I have significant reservations, as outlined above.

## References

[1] UnSCakirVKaraCTurkHKoseOBalliOYilmazY: Risk factors for hemorrhage requiring embolization after percutaneous nephrolithotomy. *Can Urol Assoc J*.9(9-10) : 10.5489/cuaj.2803 E594-8 10.5489/cuaj.2803 26425220PMC4581924
